# Effect of *Mucuna pruriens* on brain NMDA receptor and tau protein gene expression in cerebral ischemic rats

**DOI:** 10.3389/fphys.2023.1092032

**Published:** 2023-02-16

**Authors:** Prachi P. Parvatikar, S. M. Patil, Bheemshetty S. Patil, R. Chandramouli Reddy, Ishwar Bagoji, Manjunath S. Kotennavar, Sumangala Patil, Aravind V. Patil, Kusal K. Das, Swastika N. Das, Shrilaxmi Bagali

**Affiliations:** ^1^ Laboratory of Vascular Physiology and Medicine, Department of Physiology, Shri B. M. Patil Medical College, Hospital & Research Centre, BLDE (Deemed to be University), Vijayapura, Karnataka, India; ^2^ Department of Anatomy, Shri B. M. Patil Medical College, Hospital & Research Center, BLDE (Deemed to be University), Vijayapura, Karnataka, India; ^3^ Department of Biochemistry, Shri B. M. Patil Medical College, Hospital & Research Center, BLDE (Deemed to be University), Vijayapura, Karnataka, India; ^4^ Department of Surgery, Shri B. M. Patil Medical College, Hospital & Research Center, BLDE (Deemed to be University), Vijayapura, Karnataka, India; ^5^ Department of Chemistry, BLDEA’s V P Dr PG Halakatti College of Engineering and Technology, Vijayapura, Karnataka, India

**Keywords:** *Mucuna pruriens*, β-sitosterol, left common carotid artery occlusion, NMDA receptor, tau protein, brain histopathology

## Abstract

Present study aimed to assess effect of pre-treatment with *Mucuna pruriens* seed extract and its bioactive molecule(s) on NMDAR and Tau protein gene expression in cerebral ischemic rodent model. Methanol extract of *M. pruriens* seeds was characterized by HPLC, and β-sitosterol was isolated by flash chromatography. *In vivo* studies to observe the effect of pre-treatment (28 days) with methanol extract of *M. pruriens* seed and β-sitosterol on the unilateral cerebral ischemic rat model. Cerebral ischemia induced by left common carotid artery occlusion (LCCAO) for 75 min (on day 29) followed by reperfusion for 12 h. Rats (*n* = 48) divided into four groups. GroupI (control,Untreated + LCCAO)-No pre-treatment + cerebral ischemia; GroupII(β-sitosterol + Sham)-pre-treatment with β-sitosterol, 10 mg/kg/day + sham-operated; GroupIII(β-sitosterol + LCCAO)-pre-treatment with β-sitosterol, 10 mg/kg/day + cerebral ischemia; GroupIV(methanol extract + LCCAO)-pre-treatment with methanol extract of *M. pruriens* seeds, 50 mg/kg/day + cerebral ischemia. Neurological deficit score was assessed just before sacrifice. Experimental animals were sacrificed after 12 h reperfusion. Brain histopathology was performed. Gene expression of NMDAR and Tau protein of left cerebral hemisphere (occluded side) was performed by RT-PCR. Results revealed that the neurological deficit score was lower in groups III and IV compared to group I. NMDAR and tau protein mRNA expression in left cerebral hemisphere were upregulated in Group I, downregulated in groups III and IV. Histopathology of left cerebral hemisphere (occluded side) in Group I showed features of ischemic brain damage. Groups III and IV, left cerebral hemisphere showed less ischemic damage compared GroupI. Right cerebral hemisphere showed no areas of ischemia-induced brain changes. Pre-treatment with β-sitosterol and methanol extract of *M. pruriens* seeds may reduce ischemic brain injury following unilateral common carotid artery occlusion in rats.

## 1 Introduction

With its two subtypes, ischemic and hemorrhagic, stroke is among the leading causes of morbidity and mortality. Ischemic cerebral injury accounts for 87% of all stroke cases (Silva et al., 2017; Wu and Tymianski, 2018). To date, the recanalization of the occluded vessels with tissue plasminogen activator is the widely used therapeutic option. However, the narrow therapeutic time window (within 4.5 h post-stroke) and the severe tissue damage following reperfusion are significant limitations to this treatment modality (Martin and Wang, 2010).

The role of NMDA (N-methyl-d-aspartate) receptors and Tau protein in ‘excitotoxicity’, a mechanism central to neuronal death post cerebral ischemia, have been delineated (Martin and Wang, 2010). Consequently, targeting NMDA receptors and Tau protein could be potentially effective in managing ischemic stroke that can be further explored.

Medicinal plants have long been relied upon in managing several diseases, including cerebrovascular diseases. They have an important place in traditional medicine, including Ayurveda, an Indian system of medicine. The health benefits and therapeutic effects of plants are due to the properties of secondary plant metabolites (phytochemicals) they contain. Phytochemicals have been demonstrated to affect several pathophysiological processes underlying stroke like oxidative stress, inflammation and apoptotic cell death. Hence, phytochemicals are a better, safer, and cost-effective alternative to synthetic drugs (Kim et al., 2016; Hussein et al., 2018; Surguchov et al., 2021). *Mucuna pruriens* is one such medicinal plant that is widely used in Ayurveda (Pathania et al., 2020).

*Mucuna pruriens* (*M. pruriens*), routinely known as Mucuna or velvet bean, is a popular medicinal plant belonging to the legume family and common in tropical and subtropical areas worldwide. In India, it is widely found in the eastern part of the country (Kamkaen et al., 2022). Several studies have reported the anti-Parkinson, antidiabetic, antioxidant, antibacterial, antiepileptic, antineoplastic, male fertility, and aphrodisiac effects of *M. pruriens* (Rane et al., 2019; Theansungnoen et al., 2022)*.* Phytochemical analysis of *M. pruriens* seed extract has demonstrated a significant content of L-dopa accounting for its anti-parkinsonism properties. It has been widely used in Ayurveda as a medicinal plant since Vedic times and is used to treat various nervous disorders, probably due to its neuroprotective properties (Rane et al., 2019; Kamkaen et al., 2022). Hence, the primary objective of the present work was to study the possible neuroprotective effect of pretreatment with *M. pruriens* seed extract and its isolated bioactive molecule(s) in the cerebral ischemic rat model and its impact on NMDA receptor and Tau protein gene expression.

## 2 Materials and methods

### 2.1 *In vitro* studies

*In vitro* studies involved phytochemical extraction of *M. pruriens* seeds and determining the total alkaloids, flavonoids, phenols, in the seed extract. The antioxidant property of the seed extract was assayed. Further, the study involved HPLC and flash chromatography to estimate and isolate bioactive molecule(s) from *M. pruriens* seed extract.

#### 2.1.1 Phytochemical extraction

The seeds of *M. pruriens* were collected from Karnataka, India. The seed powder was subjected to extraction using methanol, ethanol, petroleum ether, chloroform, and water solvents (Senguttuvan et al., 2014; Kalebar et al., 2020). The qualitative analysis of the solvent extracts revealed that the methanol extract of *M. pruriens* seeds possessed a superior phytochemical profile additionally supported by the *in vitro* antioxidant assays (Lampariello et al., 2012; Rane et al., 2019). Hence, in the present study methanol extract of *M. pruriens* seeds was used. Methanol extract of *M. pruriens* seed will be denoted as *M. pruriens* seed extract, henceforth.

Methanol extract of *M. pruriens* seeds was subjected to determination of total alkaloids, flavonoids, phenols as per standard protocol (Senguttuvan et al., 2014; Kalebar et al., 2020). The antioxidant efficiency of *M. pruriens* seed extract was determined by hydrogen peroxide scavenging assay and 1,1-Diphenyl-2-Picrylhydrazyl (DPPH) radical scavenging assay as per standard protocol (Brand-Williams et al., 1995; Siddhuraju and Becker, 2003; Keser et al., 2012).

#### 2.1.2 HPLC and flash chromatography analysis

By using HPLC, the bioactive molecules, namely, L-DOPA and β-sitosterol were identified from *Mucuna prurines* seed extract (Kshirsagar et al., 2016). Waters HPLC system (Waters/Millipore, Milsford, MA, United States) which included a model 515 pump, a model 2,487 dual wavelength absorbance detector was employed. A C18 column was used. The mobile phase was constituted by A (water) and B (acetonitrile). The gradient range varied linearly from 50% to 90% B in 4 min with injection volume 2 mL for the RRHT column. The flow rate was 1.0 mL/min, the column temperature was maintained at 30°C, and the detection wavelength was 280 nm (Pawar et al., 2010). The extraction of bioactive molecule β-sitosterol from *M. pruriens* seed extract was done by Reversed-Phase Flash Chromatography. The isolation was carried out in Biotage Flash 75 system (Biotage, A Division of Dyax Corporation, Charlottsville, VA, United States) (Herrera et al., 2020).

### 2.2 *In vivo* study

*In vivo* study was undertaken to investigate for the possible neuroprotective effect of *M. pruriens* seed extract as well as its isolated bioactive molecule in rat model of cerebral ischemia and to understand the underlying molecular mechanism by analyzing the gene expression of brain NMDAR and Tau protein.

#### 2.2.1 Ethical approval

Ethical approval for the study was obtained from Institutional Animal Ethics Committee (IAEC) (No.08/BLDE (DU)/2021). The animal care guidelines set forth by the Committee for the Purpose and Control and Supervision of Experiments on Animals (CPCSEA), Ministry of Environment and Forests (Animal Welfare Division), Government of India were strictly adhered to throughout the study.

#### 2.2.2 Experimental animals

The study involved 48 male Wistar rats, aged 8–10 weeks, weighing 180–250 gm. The experimental animals were maintained at 22°C–24°C and a 12-h light/12-h dark cycle with food and water *ad libitum*. The animals were acclimatized to the laboratory conditions for 7 days before initiating the experimental protocol.

#### 2.2.3 Study design

The experimental animals (*n* = 48) were allocated to one of the four groups. Group I (Untreated + LCCAO) served as control. No pre-treatment. Cerebral ischemia induced (on day 29) by LCCAO for 75 min followed by reperfusion for 12 h. Group II (β-sitosterol + Sham)—Pre-treatment with β-sitosterol, 10 mg/kg/day, (Ayaz et al., 2017), oral, 28 days + sham-operated (on day 29). Group III (β-sitosterol + LCCAO) - pre-treatment with β-sitosterol, 10 mg/kg/day, oral, 28 days + cerebral ischemia induced (on day 29) by LCCAO for 75 min followed by reperfusion for 12 h. Group IV (methanol extract + LCCAO) - pre-treatment with methanol extract of *Mucuna pruriens* seeds, 50 mg/kg/day (Nayak et al., 2017), oral, 28 days + cerebral ischemia induced by LCCAO for 75 min followed by reperfusion for 12 h (on day 29).

The bodyweight of rats was measured using digital weighing machine (Practum 1102-10IN, Germany). The experimental animals in each group were matched for the body weight at the study onset. Daily food consumption of each rat was monitored during the intervention period.

#### 2.2.4 Cerebral ischemia

Cerebral ischemia was induced by left common carotid artery occlusion (LCCAO) for 75 min followed by 12 h reperfusion (Das et al., 2018). The surgical procedure for inducing cerebral ischemia was performed in the morning hours in overnight fasted rats. The animals were subjected to the procedure after the intervention period of 28 days (i.e., on day 29). Strict aseptic precautions were taken during the surgical procedure. The experimental animals were anesthetized with ketamine (60 mg/kg body weight, i. p.) and xylazine (6 mg/kg body weight). An oblique incision was taken alongside sternocleidomastoid in the neck from the base of the mandible to sternum. At the level of greater of cornu of thyroid cartilage, deep fascia and carotid sheath were incised. The common carotid artery was exposed and separated from the fascia, while protecting the vagus and jugular vein from any damage. A nylon thread (0.5 mm) was passed from behind the artery and ligated. The occlusion was maintained for 75 min following which the ligature was released and allowed for 12 h reperfusion. The deep fascia and carotid sheath were closed with proline. Superficial fascia and skin were closed with all aseptic precautions. Sham operation (group II) involved the same surgical steps except for left common carotid artery occlusion. The experimental animals were closely monitored for physiological parameters like heart rate, blood pressure and respiratory rate. Non-Invasive Blood Pressure was recorded with a tail cuff sensor (Biopac NIBP200A model). For heart rate, ECG was recorded using needle subcutaneous electrodes (Biopac Student Lab 4.1). For respiratory rate, pneumogram was recorded using respiratory pad transducer (Biopac Student Lab 4.1). Body temperature was maintained at 36°C–37.5°C using a warm pad. The surgery was uneventful and all the rats recovered following surgery.

#### 2.2.5 Neurological deficit score

Post-ischemic neurological deficit was assessed after 12 h of reperfusion and before sacrifice using a six point scale neurological score: 0 = no neurological deficit; 1 = failure to extend contralateral forelimb fully on lifting the animal by tail; 2 = circling to the contralateral side; 3 = falling to the contralateral side; 4 = no spontaneous walk or in a comatose state; 5 = death (Komatsu et al., 2021).

#### 2.2.6 Analysis of brain NMDA receptor and tau protein gene expression

The experimental animals were sacrificed (*n* = 6/group) after 12 h reperfusion. The brain was carefully dissected. For gene expression analysis, the left cerebral hemisphere (occluded side) was used. Tissue was immediately stored in RNAlater at −80°C for RT-PCR analysis.

NMDA receptor, tau protein and β-actin (endogenous control) mRNA expressions were assessed by using quantitative reverse transcription-PCR (RTPCR). RNeasy kit was used to extract RNA from left cerebral hemisphere homogenates as per the manufacturer’s protocol. Reverse transcription of RNA was done using SuperScript II Reverse Transcriptase as per the prescribed protocol. cDNA was amplified with quantitative reverse transcription-PCR (Kong et al., 2021). The gene expression of NMDAR and Tau protein are presented as fold change calculated by using the formula 2^-ΔΔCq (Herur et al., 2022). PCR primers specific to each gene are as depicted in Table 1. The PCR conditions are provided as Supplementary Table S1.

**TABLE 1:** Primer pairs used for quantitative RT-PCR analysis.

	Direction	Primer	Amplicon size (bp)
NMDA receptor	Forward	TGC​ATC​TAT​GAT​CAT​GGC​TGA​C	206
	Reverse	ATA​AAG​CTG​ATG​AAG​TCT​CGG​TAG-	
Tau Protein	Forward	CGG​CGT​AAG​CAA​AGA​CA-3′	65
	Reverse	TGTAGCCGCTTCGTTCT	
β-actin (House keeping)	Forward	TAC​AAC​CTC​CTT​GCA​GCT​CC	120
	Reverse	TAC​AAC​CTC​CTT​GCA​GCT​CC	

#### 2.2.7 Histopathological examination

The animals (*n* = 6/group) were sacrificed after 12 h reperfusion. Brain was carefully dissected and initially fixed in bouin’s fluid and shifted to 10% neutral buffered formalin 24 h later. For histopathological examination, 2–3 mm thick slices of cerebral hemispheres were made. Bilateral blocks of the brain were embedded in paraffin wax and 2.0 to 3.0 μ sections were cut on a rotary microtome and stained with hematoxylin and eosin (H and E). Blocks included sections from the body of the corpus callosum with coronal section of white matter, splenium of the corpus callosum, posterior limb of the internal capsule with adjacent thalamus, and rostral pons. The stained sections were examined under light microscope (Olympus CH20i) with Samsung Digital Color Camera (Model No. SDC-242, N. J 07094, United States).

## 3 Statistical analysis

SPSS software (version 20.0) was used for statistical analysis. Data are presented as Mean ± SD. To analyze statistical significant difference across multiple groups, one way ANOVA was used followed by Tukey’s *post hoc* test to ascertain significant intergroup differences. *p* < 0.05 was considered statistically significant.

## 4 Results

### 4.1 *In vitro* studies

In the present study the pharmacognostical and phytochemical profile of *M. pruriens* was screened by sequential phytochemical extraction from its seed powder using different solvents *viz.*, methanol, ethanol, petroleum ether, chloroform, and water. The qualitative analysis of the phytochemical constituents of *M. pruriens* seed extract with different solvents is provided as Supplementary Table S2. The phytochemical constituents in *M. pruriens* seed extract is provided as Supplementary Tables S3, S4. The antioxidant activity was evaluated by H_2_O_2_ scavenging assay and DPPH radical scavenging assay. The results of DPPH radical scavenging assay and H_2_O_2_ scavenging assay are provided as Supplementary Tables S5, S6. The DPPH and hydrogen peroxide scavenging activity of methanol extract of *M. pruriens* seeds was concentration dependent with greater scavenging at higher concentration. Ascorbic acid was used as a control and the scavenging activity of ascorbic acid was similarly concentration dependent.

#### 4.1.1 HPLC and flash chromatography

**FIGURE 1:**
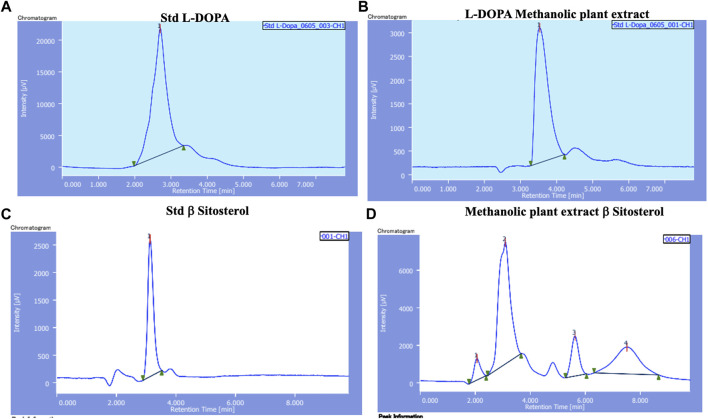
HPLC chromatograms of **(A)** L-DOPA standard; **(B)** L-DOPA from methanol extract of *M. pruriens* seed; **(C)** β-sitosterol standard; **(D)** β-sitosterol from methanol extract of *M. pruriens* seed.

HPLC chromatograms of standard solutions of L-DOPA and β-sitosterol and methanol extract of *M. pruriens* are presented in Figure 1. The calibration curve showed good linearity with correlation coefficients (R^2^- 0.9975). Based on the data, the estimates of L-DOPA and β-sitosterol in the *M. pruriens* seed extract was 20mg/100 gm and 12mg/100 gm respectively.

### 4.2 *In vivo* studies

#### 4.2.1 Physiological parameters

The comparison of physiological parameters before and after surgery as presented as Supplementary Table S7. Heart rate, SBP, DBP and respiratory rate were similar between groups before surgery. After surgery there was an increase in heart rate, SBP, DBP and respiratory rate across all groups. Heart rate and SBP were higher in group I (control) compared to other groups.

#### 4.2.2 Neurological deficit score

Table 2 presents comparison of neurological deficit scores among groups. Neurological deficit score was higher in group I (Control, Untreated + LCCAO), group III (β-sitosterol + LCCAO) and group IV (methanol extract + LCCAO) compared to group II (β-sitosterol + Sham). The scores were lower in group III (β-sitosterol + LCCAO) and IV (methanol extract + LCCAO) compared to group I (Control, Untreated + LCCAO) although not statistically significant.

**TABLE 2:** Neurological deficit score of experimental animals.

	Group I (control, Untreated + LCCAO)	Group II (β-sitosterol + sham)	Group III (β-sitosterol + LCCAO)	Group IV (methanol extract + LCCAO)	ANOVA	
					F	P
Neurological deficit score	4.5 ± 1.64^a^	0 ± 0^b^	4.0 ± 1.54^a^	4.0 ± 154^a^	14.06	0.000*

Superscripts a, b, indicate significant difference between groups. *p ≤ 0.05. *n* = 6 per group.

#### 4.2.3 NMDAR and tau protein gene expression in left cerebral hemisphere homogenate

Table 3 presents Real time PCR results for NMDAR and Tau protein mRNA expression in experimental animals. mRNA expression of NMDAR and Tau protein was higher in Group I (control,Untreated + LCCAO) compared to group II (β-sitosterol + sham). In the pretreated groups, group III (β-sitosterol + LCCAO) and group IV (methanol extract + LCCAO) mRNA expression of NMDAR and Tau protein was lower compared to group 1 (control, Untreated+LCCAO).

**TABLE 3:** Gene expression of NMDAR and Tau protein in left cerebral hemisphere homogenate.

	Group I (control, Untreated + LCCAO)	Group II (β-sitosterol + sham)	Group III (β-sitosterol + LCCAO)	Group IV (methanol extract + LCCAO)
NMDA receptor	1.5	1.00	1.28	1.23
Tau Protein	1.4	1.00	1.31	1.27

*n* = 6/group.

#### 4.2.4 Histopathological examination of cerebral hemispheres

**FIGURE 2:**
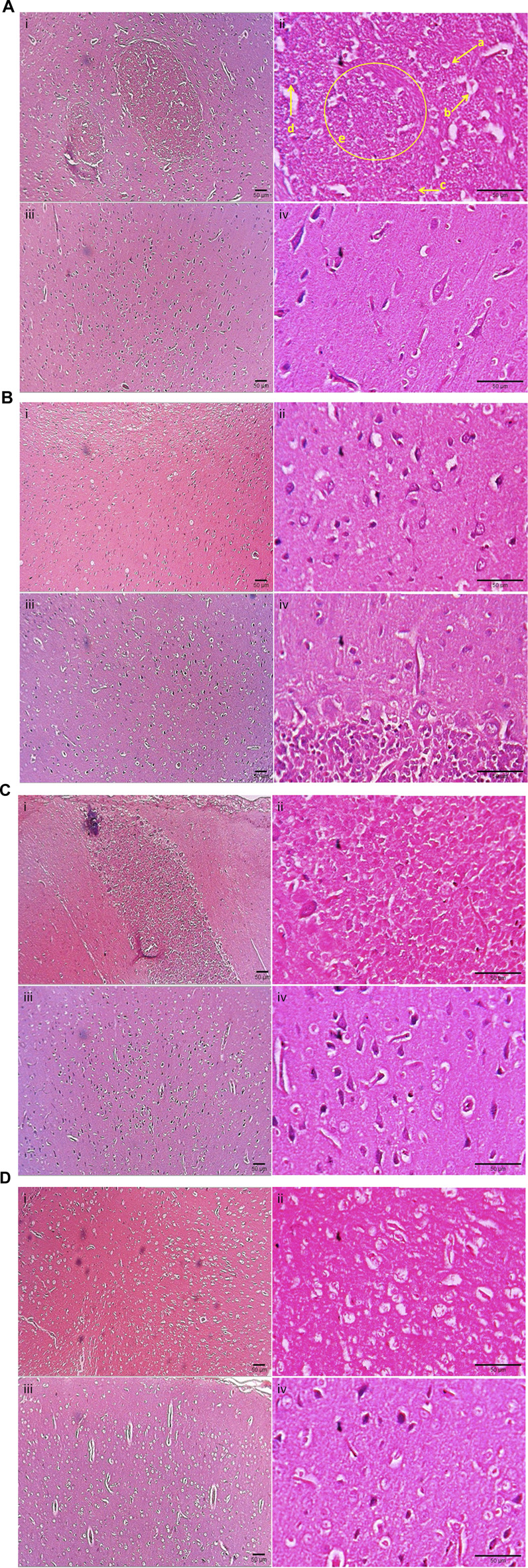
Photomicrograph depicting cerebral cortices stained with hematoxylin and eosin of Groups I, II, III, IV. **(A)**: Photomicrograph of cerebral cortices stained with hematoxylin and eosin of Group I (control, Untreated + LCCAO) showing; i) Left (occluded) cerebral cortex (×10), ii) Left (occluded) cerebral cortex (×40), iii) Right (non-occluded) cerebral cortex (×10), iv) Right (non-occluded) cerebral cortex (×40). a - Pyknotic nuclei, b – red neurons with purkinje fibre, c- mild lymphocytic infiltration, d - prominent stained nuclear component, e - multiple hazy nuclei with increased intercapsular space. **(B)**: Photomicrograph of cerebral cortices stained with hematoxylin and eosin of Group II (β-sitosterol + Sham) showing: i) Left (occluded) cerebral cortex (×10), ii) Left (occluded) cerebral cortex (×40), iii) Right (non-occluded) cerebral cortex (×10), iv) Right (non-occluded) cerebral cortex (×40). **(C)**: Photomicrograph of cerebral cortices stained with hematoxylin and eosin of Group III (β-sitosterol + LCCAO) showing; i) Left (occluded) cerebral cortex (×10), ii) Left (occluded) cerebral cortex (×40), iii) Right (non-occluded) cerebral cortex (×10), iv) Right (non-occluded) cerebral cortex (×40). **(D)**: Photomicrograph of cerebral cortices stained with hematoxylin and eosin of Group IV (methanol extract + LCCAO) showing; i) Left (occluded) cerebral cortex (×10), ii) Left (occluded) cerebral cortex (×40), iii) Right (non-occluded) cerebral cortex (×10), iv) Right (non-occluded) cerebral cortex (×40).

Figure 2 presents the photomicrograph of cerebral cortices stained with H&E. In Group I (control, Untreated + LCCAO), left cerebral hemisphere (occluded side) showed cerebral edema (gross alteration), altered microscopic architecture with scattered cellular layers of grey matter and white matter. Neuronal damage (red neurons) with eosinophilic cytoplasm and pyknotic nuclei with a hazy appearance were noticed. Localized cystic infarcts with features of diffuse cerebral infarction were seen (Figure 2A - i, ii). There were no microscopic alterations in the left cerebral hemisphere of group II (β-sitosterol + Sham) (Figure 2B - i, ii). In Group III (β-sitosterol + LCCAO) (Figure 2C - i, ii) and IV (methanol extract + LCCAO) (Figure 2D - i, ii), left cerebral hemisphere (occluded side) showed less ischemic changes compared to group I (control, LCCAO). Right cerebral hemisphere of the brain (non-occluded side) showed no evidence of ischemic changes in all groups (Figures 2A-D - iii, iv). However, mild increase in intracellular space was noticed with no evidence of changes in corticomedullary junction in group I (Figure 2A - iii, iv).

## 5 Discussion

Brain tissue is highly vulnerable to ischemia. Even brief ischemia can trigger a complicated chain of events that could eventually lead to neuronal death (Woodruff et al., 2011). Excitotoxicity is one of the earliest discovered and widely recognized molecular mechanisms of post-ischemic neuronal damage and death. Glutamate and NMDAR (N-methyl-D-aspartate receptor) have an essential role in this mechanism (Shen et al., 2022). Additionally, the role of tau protein in excitotoxicity has been reported (Chen and Jiang, 2019).

Reduced cerebral blood flow during ischemia deranges the normal ionic homeostasis of neurons. The deranged ionic homeostasis depolarizes the neurons initiating a train of events, including the release of excitatory neurotransmitter glutamate into the synaptic cleft while impairing its re-uptake. Consequently, excess glutamate in the extracellular space causes the over-activation of NMDARs on the post-synaptic neurons (Wu and Tymianski, 2018). The excessively activated NMDARs allow uncontrolled Ca^2+^ influx causing calcium overload and triggering downstream pro-death signalling events that include activation of calpain, generation of reactive oxygen species (ROS), and mitochondrial damage, causing cell necrosis or apoptosis (Martin and Wang, 2010; Wu and Tymianski, 2018). Additionally, excitotoxicity causes tau hyperphosphorylation, significantly reducing its biological activity. There are reports of the critical role of tau in eliciting excitotoxicity. However, the molecular mechanisms underlying tau-induced excitotoxicity are yet to be elucidated (Chen and Jiang, 2019). Given the pivotal role of NMDARs and tau protein in mediating neuronal death in ischemic stroke, interventions directed at NMDAR and tau-mediated pathology could serve as a clinically beneficial approach in the prevention/management of ischemic stroke.

The present study aimed to evaluate the neuroprotective effect of pre-treatment with *M. pruriens* seed extract and its isolated bioactive molecule, β-sitosterol, in experimental animals subjected to cerebral ischemia by LCCAO. Additionally, the study explores the underlying molecular mechanisms of cerebral ischemia that include expression of NMDAR and Tau protein in experimental cerebral ischemia with or without pre-treatment with *M. pruriens* seed extract and its isolated bioactive molecule, β-sitosterol.

The current research involved *in vitro* and *in vivo* studies. *In vitro* studies aimed to screen the pharmacognostical and phytochemical profile of *M. pruriens*. For this, the shade-dried seeds of *M. pruriens* were subjected to sequential phytochemical extraction using different solvents, *viz.*, methanol, ethanol, petroleum ether, chloroform, and water. The qualitative phytochemical analysis showed the presence of alkaloids, flavonoids, terpenoids, steroids, tannins, saponins and glycosides. Of the different solvents used for phytochemical extraction the methanol extract of *M. pruriens* seeds had a better profile than others. Hence, the present study proceeded with the methanol extract of *M. pruriens* seeds. The antioxidant activity of *M. pruriens* seed extract was demonstrated *in vitro* by its ability to scavenge DPPH radicals and hydrogen peroxide. Further, *M. pruriens* seed extract was subjected to HPLC and flash chromatography to identify and isolate its bioactive molecules. L-DOPA and β-sitosterol were identified as the predominant ones. L-DOPA is a predominant constituent in *M. pruriens* seed and has been used in neurodegenerative diseases like Parkinson’s disease (Rai et al., 2020). β-sitosterol was the next predominant bioactive molecule, estimated at approximately 12mg/100 gm. The present study aimed to explore the role of β-sitosterol in cerebral ischemia-induced neuropathology. β-sitosterol, a bioactive phytosterol, is naturally present in plant cell membranes with a chemical structure similar to mammalian cell-derived cholesterol (Babu and Jayaraman, 2020). β-sitosterol possesses an array of biological functions including antioxidant, antimicrobial, angiogenic, immunomodulatory, antidiabetic, anti-inflammatory, anticancer, and antinociceptive activities (Bin Sayeed et al., 2016).

The results of *in vivo* study revealed that pretreatment with *M. pruriens* seed extract and its specific bioactive molecule, β-sitosterol improved neurological deficit score, decreased ischemic brain damage and reduced the gene expression of NMDAR and tau protein in cerebral ischemia induced by LCCAO in experimental animals. The neuroprotective effects of *M. pruriens* seed extract and its isolated bioactive molecule, β-sitosterol, can be attributed to the downregulation of NMDAR and tau protein gene expression and the downstream signalling pathways, particularly the reactive oxygen species generation. There is increasing evidence that phytochemicals regulate gene expression by modulating the transcriptional activity of genes *via* specific transcriptional factors or epigenetic mechanisms (Surguchov et al., 2021). A previous study by Fung et al. (2014) demonstrated a cardioprotective effect of pretreatment with *M. pruriens* seed extract against experimental Naja Sputatrix envenomation due to upregulation of genes related to energy production and metabolism, nervous system, inflammatory response, and apoptosis, among several others in the rat heart. Additionally, the neuroprotective effect of *M. pruriens* seed extract and β-sitosterol may be partly attributed to their potent antioxidant properties. Since the brain has high oxygen consumption, it is susceptible to the damaging effects of reactive oxygen species generated during ischemia-reperfusion (Lalkovičová and Danielisová, 2016). Studies have reported that pre-treatment with synthetic antioxidants decreased the infarct size in animals subjected to ischemia/reperfusion injury (Duong et al., 2008).

Interestingly, the methanol extract of *M. pruriens* seeds was more effective than its isolated bioactive molecule, β-sitosterol. Medicinal plant extract may be superior to an equivalent dose of an isolated bioactive molecule. Plant extract contains hundreds or even thousands of different bioactive molecules in variable concentrations that may contribute to its medicinal value. The interaction between these multiple compounds may be synergistic, additive or antagonistic, which may be responsible for the overall activity of the medicinal plant extract (Vaou et al., 2022). L-DOPA was a predominant phytochemical in the seed extract of *M. pruriens*. L-DOPA might have contributed to better neuroprotection offered by *M. pruriens* seed extract compared to its isolated bioactive molecule β-sitosterol.

A significant neuroprotective activity of methanol extract of *M. pruriens* seeds and its isolated bioactive molecule, β-sitosterol suggest a potential for their use in the prevention/reducing the cerebral ischemia induced neuropathology.

## 6 Conclusion

Pre-treatment with methanol extract of *M. pruriens* and its bioactive molecule β-sitosterol confers a protection against cerebral ischemic injury by down regulating the gene expression of NMDAR and tau protein that mediate excitotoxicity.

## Data Availability

The raw data supporting the conclusion of this article will be made available by the authors, without undue reservation.
