# Supplementary material for: Effect of Mucuna pruriens on brain NMDA receptor and tau protein gene expression in cerebral ischemic rats
Source: Front Physiol. 2023 Feb 16;14:1092032. doi: 10.3389/fphys.2023.1092032 (PMC9978338; doi:10.3389/fphys.2023.1092032)
Supplement: Supplementary file 1 [file Table1.DOCX]

Supplementary File

Table S1: PCR specifications

| mRNA | PCR reaction condition |  |
| --- | --- | --- |
| NMDA | 94°C for 90 seconds, 60°C for 45 seconds, and 72°C for 45 seconds for 35 cycles | 72°C 10min  4°C stop reaction |
| Tau protein | 94°C for 100 seconds, 60°C for 40 seconds, and 72°C for 40 seconds for 32 cycles. |  |
| β-actin | 94°C for 90 seconds, 60°C for 45 seconds, and 72°C for 45 seconds for 35 cycles |  |

**Table S2**: Qualitative analysis of Phytochemicals in *M. Prurines* seed extract with different solvents

| Phytochemical constituents | Chloroform | Petroleum ether | Methanol | Ethanol | Water |
| --- | --- | --- | --- | --- | --- |
| Alkaloids | + | ++ | ++ | ++ | ++ |
| Flavonoids | ++ | ++ | ++ | ++ | ++ |
| Glycosides | -- | + | + | + | ++ |
| Saponins | -- | -- | -- | -- | ++ |
| Steroids | -- | ++ | ++ | ++ | ++ |
| Tannin | -- | + | + | + | ++ |
| Terpenoids | + | ++ | ++ | ++ | ++ |

**Table S3**: Phytoconstituents of *M. Prurines* seed extract

| **Phytoconstituents** | mg/ml |
| --- | --- |
| Moisture | 10.6 |
| Ash | 4.4 |
| Protein | 10 |
| Lipid | 9.3 |
| Carbohydrate | 14 |
| Crude fibre | 15.4 |

**Table S4: Phytochemical content in plant extract**

| **Phytoconstituents** | **Standard** | **Concentration (mg/ml)** |
| --- | --- | --- |
| Phenol | Gallic acid | 4.90 |
| Tannin | Tanninc acid | 10.40 |
| Flavonoid | Quercetin | 17.34 |
| Alkaloid |  | 0.98 |

**Table S5: 2, 2-Diphenyl-1-picrylhydrazyl radical scavenging ability (DPPH) assay**

| **Concentration**  **(µg/ml)** | **% Scavenging activity** | |
| --- | --- | --- |
|  | **Ascorbic acid** | ***Methanol extract of M. pruriens* seeds** |
| **20** | 35.06 ± 0.01 | 40.5± 0.01** |
| **40** | 46.03 ± 0.01 | 46.4± 0.01 |
| **60** | 54.05 ± 0.01 | 50.1± 0.01** |
| **80** | 67.12 ± 0.02 | 52.03 ± 0.01** |
| **100** | 76.03 ± 0.03 | 66.54 ± 0.01** |

Data is presented as mean standard error of the mean (n = 3). The values were significantly different when compared to the standard at the same concentration (standard-ascorbic acid) ** p < 0.01.

**Table S6: Hydrogen Peroxide Scavenging Activity**

| Concentration  (**µg/ml**) | % Scavenging activity | |
| --- | --- | --- |
|  | **Ascorbic acid** | ***Methanol extract of M. pruriens* seeds** |
| 20 | 29.82 ± 0.01 | 28.96± 0.01 |
| 40 | 36.7 ± 0.01 | 35.8± 0.01 |
| 60 | 39.6 ± 0.01 | 40.7± 0.01 |
| 80 | 44.5 ± 0.02 | 42.60± 0.01 |
| 100 | 47.03 ± 0.03 | 47.00 ± 0.01 |

Data is presented as mean ± standard error of the mean (n = 3). Values not significantly different when compared to the standard (ascorbic acid)

Table S7: Comparison of physiological parameters before and after surgery

|  | Group I  (Control, Untreated+  LCCAO ) | Group II  (β-sitosterol + Sham) | Group III  (β-sitosterol+ LCCAO) | Group IV  (methanol extract + LCCAO) | ANOVA | |
| --- | --- | --- | --- | --- | --- | --- |
|  |  |  |  |  | F | P |
| Heart Rate  (Before surgery) | 274.66±11.75^a^ | 268.16±14.52^a^ | 283.83±13.27^a^ | 278.50±17.51^a^ | 1.25 | 0.317 |
| Heart Rate  (After surgery) | 309.16±8.99^a^ | 287.33+15.73^b^ | 275.83+11.82^b^ | 272.50+15.41^b^ | 10.17 | 0.000* |
| SBP (mm Hg)  (Before surgery) | 108.0+7.48^a^ | 99.16+6.01^a^ | 103.83+4.66^a^ | 99.00+7.45^a^ | 2.61 | 0.079 |
| SBP (mm Hg) (After surgery) | 113.66+5.71^a^ | 102.50+7.58^b^ | 103.33+6.53^b^ | 103.00+3.74^b^ | 4.71 | 0.012* |
| DBP (mm Hg) (Before surgery) | 70.83+3.48^a^ | 72.16+2.85^a^ | 73.00+3.74^a^ | 70.16+3.48^a^ | 0.845 | 0.486 |
| DBP (mm Hg) (After surgery) | 76.33+2.73^a^ | 74.33+3.50^a^ | 71.83+3.71^a^ | 74.66+2.16^a^ | 2.170 | 0.123 |
| Respiratory Rate (cycles/min)  (Before surgery) | 14.66+2.16^a^ | 13.00+1.41^a^ | 13.33+2.16^a^ | 13.50+1.87^a^ | 0.850 | 0.483 |
| Respiratory Rate (cycles/min)  (After surgery) | 15.00+2.36^a^ | 16.50+1.37^a^ | 14.50+2.42^a^ | 14.66+1.63^a^ | 1.245 | 0.320 |

Superscripts a, b indicate significant difference between groups. **P*<0.05. n=6 per group.
